# *Vitreoscilla* Haemoglobin: A Tool to Reduce Overflow Metabolism

**DOI:** 10.3390/microorganisms10010043

**Published:** 2021-12-26

**Authors:** Hilal Taymaz-Nikerel, Alvaro R. Lara

**Affiliations:** 1 Department of Genetics and Bioengineering, Istanbul Bilgi University, İstanbul 34060, Turkey; hilal.nikerel@bilgi.edu.tr; 2 Departamento de Procesos y Tecnología, Universidad Autónoma Metropolitana, Mexico City 05348, Mexico

**Keywords:** overflow metabolism, P/O ratio, *Vitreoscilla* haemoglobin, flux balance analysis

## Abstract

Overflow metabolism is a phenomenon extended in nature, ranging from microbial to cancer cells. Accumulation of overflow metabolites pose a challenge for large-scale bioprocesses. Yet, the causes of overflow metabolism are not fully clarified. In this work, the underlying mechanisms, reasons and consequences of overflow metabolism in different organisms have been summarized. The reported effect of aerobic expression of *Vitreoscilla* haemoglobin (VHb) in different organisms are revised. The use of VHb to reduce overflow metabolism is proposed and studied through flux balance analysis in *E. coli* at a fixed maximum substrate and oxygen uptake rates. Simulations showed that the presence of VHb increases the growth rate, while decreasing acetate production, in line with the experimental measurements. Therefore, aerobic VHb expression is considered a potential tool to reduce overflow metabolism in cells.

## 1. Overflow Metabolism and Bioprocessing

Fast growing cells often display incomplete oxidation of the carbon source, even if oxygen is present in non-limiting amounts. As a result, partially oxidized molecules, rather than CO_2_, are excreted to the environment. This phenomenon was already observed for yeasts by Louis Pasteur in 1861 [1] and later on in muscle cells [2], carcinoma cells [3] and normal tissues after viral infection [4]. Traditionally referred to as the “Pasteur”, “Warburg” and “Crabtree” effect, such a metabolic state is collectively known as overflow metabolism [5].

The biotechnological production of molecules requires the transformation of a substrate (most commonly glucose) into the desired molecule. Because these are autocatalytic processes, the amount of product that can be synthesized depends on the amount of cells in the culture. Therefore, a standard procedure to maximize the amount of synthesized product is to attain an elevated amount of cells in the culture. These so-called high cell-density cultures require elevated amounts of carbon source that lead to overflow metabolism.

Examples of overflow metabolites excreted by organisms of biotechnological relevance are shown in Table 1. The accumulation of such by-products lowers the pH of the broth, affecting the cellular physiology. The continuous addition of alkali to control pH can result in the accumulation of ions and osmolality increase that can negatively affect the cells. Moreover, the formation of overflow metabolites can be seen as a waste of carbon that otherwise could have been incorporated to biomass and/or product. For instance, the amount of acetate produced as overflow metabolite can be as high as 15% (*w/w*) of the carbon source (glucose) consumed [6].

Diverse strategies have been applied to reduce overflow metabolism in different organisms, including genetic interventions [7] and the slow addition of the carbon source (fed-batch cultures) [8]. Nevertheless, all the proposed solutions also imply disadvantages. Furthermore, despite the relevance of overflow metabolism from a physiological and biotechnological standpoint, its causes are not clearly understood. Some possible explanations are briefly presented below.

### 1.1. Causes of Overflow Metabolism

#### 1.1.1. Metabolic Imbalance

**Figure 1:**
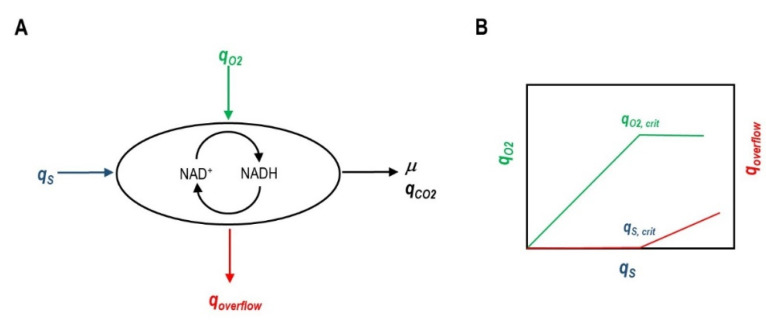
Overview of Overflow Metabolism as Originated from a Metabolic Imbalance. (**A**): NADH regeneration rate can be insufficient for given substrate and oxygen uptake rates (*q_S_* and *q_O_*_2_, respectively) to fully oxidize the carbon source to CO_2_ (*q_CO_*_2_). Therefore, overflow metabolites are produced (*q_overflow_*) to contribute to NADH regeneration. (**B**): Initially *q_O_*_2_ displays a linear correlation with *q_S_*. However, at some point *q_O_*_2_ reaches a maximum (*q_O_*_2_, *_crit_*) and *q_S_* continues increasing, with the concomitant production of overflow metabolites.

A relatively straightforward explanation of overflow metabolism considers that a metabolic imbalance between catabolism and anabolism occurs at high glucose uptake rates (*q_S_*) [17]. At low *q_S_*, the substrate can be fully oxidized via the tricarboxylic acid cycle (TCA) and NADH regenerated by the electron transport chain (Figure 1). Increasing *q_S_* is accompanied by an increased oxygen consumption rate (*q_O_*_2_). After a certain threshold value of glucose uptake (*q_s,crit_*), overflow metabolites start to accumulate. This coincides with a maximum rate of oxygen consumption (*q_O_*_2, max_). Above this respiratory capacity, *q_S_* can continue to increase, with the concomitant synthesis of overflow metabolites (for example, for *E. coli* cultures, see [18]). NADH regeneration rate could not be reached by the electron transport chain only, and therefore, fermentative pathways are activated. TCA activity can also be lowered [19]. Enzyme capacity constraints combined with flux balance analysis using a genome-scale model predicted the lactate shift in CHO cells coincident with a plateau in the oxidative phosphorylation flux and specific CO_2_ formation rate (*q_CO_*_2_) patterns in relation to *q_S_* [20].

Maintaining the cells growing at *q_S_* < *q_S,crit_* in fed-batch cultures avoids overflow metabolism and allows attaining high cell-densities. This principle has been successfully applied to a variety of microbial and animal cells [21]. Decreasing *q_S_* by genetic manipulation has also been a successful strategy to reduce overflow metabolism in *E. coli* [22,23,24,25] and CHO cells [26]. Although overflow metabolism can be completely suppressed by this approach, growth rate (*μ*) is also affected. The activity of the tricarboxylic acid cycle (TCA) in *E. coli* was increased to better cope with the high glycolytic flux at elevated *q_S_*, which decreased acetate formation [27]. Another approach to overcome overflow metabolism considering a metabolic imbalance was to increase the NAD^+^ regeneration rate. For instance, Vemuri and co-workers expressed a water-forming heterologous dehydrogenase in *E. coli* and *S. cerevisiae* [19,28]. Despite the strong reduction of overflow metabolism, a decrease on biomass yield was observed, which is probably linked to the waste of reductive power to form water instead of contributing to a proton gradient. The metabolic causes of overflow metabolism in mammalian cells can be more complex. For instance, Bulté and co-workers [29] proposed that pyruvate transport to the mitochondria could be a limiting factor for its complete oxidation, leading to lactate synthesis in the cytoplasm. The researchers enhanced pyruvate transport by overexpressing a mitochondrial pyruvate carrier in CHO cells, which resulted in up to 50% decrease of aerobic lactate production.

The approach of metabolic imbalance can partially explain the causes of overflow metabolism and inspire some genetic interventions to reduce it. However, other hypotheses have been proposed from different perspectives, as explained below.

#### 1.1.2. Proteome Allocation

Peebo and co-workers [30] analysed the proteome of *E. coli* growing at different *μ*. They found that as *μ* increased, the abundance of proteins related to carbohydrate transport and metabolism lowered, while those related to translation increased. In order to quantitatively describe the effects on the proteome, the authors defined the investment of translational capacity as the “protein expression cost” (defined as the product of the protein concentration multiplied by its length in amino acids). The expression cost of the ATP synthase and NADH dehydrogenase I relative to the total proteome increased proportionally to *μ* and reached a plateau coincident with the shift to overflow metabolism. Although the authors did not clearly link these results with the control of overflow metabolism, they suggested that *E. coli* shifts to a more economic protein usage. Due to the high demand of protein resource for the respiratory chain (ATP synthase requiring up to 2.5% of the total translational capacity), different mechanisms for energy production are preferred. This hypothesis was analysed in detail by Basan and co-workers using *E. coli* as a model organism [31]. The authors described that, although respiration is more efficient to generate energy than fermentation, the proteome cost of the former is much higher than for the latter. For glycolytic carbon sources, the authors calculated that the proteome efficiency of energy biogenesis is approx. 750 mM ATP/A_600nm_/h for fermentation, while for respiration it was approx. 390 mM ATP/A_600nm_/h. Therefore, the authors proposed that overflow metabolism is a programmed global response of the cells to cope with the proteome demands for energy generation. Further modelling and flux balance analysis have been applied to predict overflow metabolism in *E. coli* [32]. Chen and Nielsen [33] also modelled the energy metabolism of *E. coli* and *S. cerevisiae* coupled to proteomic analysis to successfully predict the start of overflow metabolism in relation to *μ*, *q_S_*, or ATP rate. Interestingly, they found that the differences between energy yields of respiration and fermentation were much larger for *E. coli* than for *S. cerevisiae*. Proteome allocation coupled with dynamic flux balance analysis and adjustable maintenance energy level allowed good prediction of growth overflow metabolism and recombinant protein production of engineered *E. coli* strains [34].

The hypothesis of proteome allocation as the origin of overflow metabolism has also been evaluated for *Lactococcus lactis* [35] and *Clostridium ljungdahlii* [36]. Proteome reduction to develop minimal cells has been reported for *E. coli* [37]. Proteome-reduced *E. coli* strains have a superior performance for plasmid DNA vaccines production in batch and fed-batch mode [38]. However, overflow metabolism in proteome-reduced cells has not yet been thoroughly tested.

#### 1.1.3. Molecular Crowding

It has been suggested that cells have evolved to maintain the enzyme-protein levels at the minimum level compatible with function. The volume occupied by proteins in the cell is 20–30% of the cell volume (determined for bacterial, yeast and mammalian cells). This large proportion may limit the diffusion and solubility of molecules in the cell due to the viscosity of the remaining unbound water [39]. Therefore, it is possible that not only the efficiency to produce ATP, but also the amount of proteins (and the volume) needed for a given pathway may be key for the cell to choose a particular energy-generation mechanism [40]. This hypothesis has been tested using modelling and experimental measurements. A flux balance model of the metabolism of *E. coli* including the constraint for the concentration of enzymes, named “FBA with Molecular Crowding” (FBAwMC) was introduced by Beg and co-workers [41]. This model could predict the sequence of utilization of carbon sources in mixtures, as well as *μ*. Although overflow metabolism was also predicted, the model estimated a lesser excretion of acetate than the experimentally determined. FBAwMC was refined and combined with enzyme activity measurements to analyze metabolic shifts from low to high *μ* in *E. coli* [42]. Vazquez et al. [43] applied FbwMC to simulate the overflow metabolism of murine LS and hybridoma cells. The shift to lactate production and the threshold *q_S_* values were estimated with good accuracy. According to their calculations, the mitochondria contribute 5 times more to molecular crowding than glycolytic enzymes and 50 times more than lactate dehydrogenase. Van Hoek and co-workers used FBAwMC to study the metabolism of *L. lactis* and S. cerevisiae yielding interesting results [44]. In agreement with previous independent reports, the authors concluded that the shift to lactate production is determined by the existence of a limited cytoplasmic solvent capacity for allocating the components of the ATP generation pathways.

Important elements of the energy production cellular machinery are located in the membranes. Therefore, molecular crowding of the membrane can be a limiting factor to shift from purely aerobic to aerobic-fermentative energy production. For example, crowding of the cytochromes in the membrane of *E. coli* was introduced as an additional constraint in FBA [45]. The well-known differential use of cytochromes depending on *q_S_* and oxygen availability was well simulated. However, the *q_S,crit_* obtained did not agree with the experimental data. This could be probably due to the fact that other important molecules, such as ATPase were not considered. In a more comprehensive study, Szenk et al. [46] integrated the *μ*-dependent surface-to-volume ratio, as well as the physical size and of the proteins involved in respiration and fermentation, to evaluate whether pure respiration could be limited by membrane crowding at fast *μ*. According to their calculation, the surface efficiency (given in ATP/s/nm^2^) for aerobic respiration is three, while it reaches a value of 15 for acetate fermentation. Furthermore, they found that the percentage of the membrane occupied by the electron transport chain component increases with the growth rate, and plateau at a value of ca. 8%, coincident with the onset of overflow metabolism. Moreover, the authors linked the surface efficiency with the phosphate/oxygen (P/O, depending on the molecules of ATP produced per NADH equivalent) ratio. P/O ratio decreases when the surface efficiency increases (fermentation), and the growth rates at which overflow metabolism triggers are higher at lower P/O ratios.

## 2. *Vitreoscilla* Haemoglobin as a Tool to Reduce Overflow Metabolism

### 2.1. Aerobic Expression of Vitreoscilla Hemoglobin

The haemoglobin of the aerobic bacteria *Vitreoscilla stercoraria* (VHb) is a single domain haemoglobin that exists as a dimeric protein of two identical subunits with a mass of 15.7 Da each [47]. Initially identified as a cytochrome, evidence was provided showing that VHb can pump Na^+^ transmembranally, instead of protons [48]. VHb displays a remarkably high capacity for oxygen delivery to terminal oxidases (high dissociation rate *k_off_* = 78 mM^−1^ s^−1^) [47]. In consequence, VHb has been used as a strategy to improve cellular performance, particularly under oxygen-limited environments, enhancing the *q_O_*_2_ and *μ* and biomass formation (for reviews see [47,49,50,51,52]). The impact of VHb expression is ample and some features depend on the host. The effect of VHb has been mostly characterized in *E. coli* cells. In *E. coli*, VHb expression restored the oxygen consumption in a strain lacking both cytochromes (Cyo and Cyd), reaching 70% of the respiration rate of the wild type strain [53], and is physiologically active in the oxygen-carrying form during aerobic respiration [54]. The H^+^/O ratio, the transmembrane ΔpH, and the ATP content of VHb-expressing *E. coli* cells were 1.5, 1.6 and 2 times greater, respectively, than the corresponding values in non-expressing cells [55]. VHb-expressing *E. coli* cells displayed higher oxidative activity than non-expressing cells, as indicated by the Redox Sensor Green fluorescence [56].

Cellular localization of VHb has also been investigated, due to its relationship with the respiration proteins. Khosla and Bailey [57], using fractionation and proteinase K accessibility techniques, determined that nearly 40% of VHb is in the periplasmic space in *E. coli*. In contrast, Ramandeep and co-workers [58], using immunogold labelling reported that more than 90% of the VHb was located in the periplasm of *E. coli*, and that 57% of the VHb was localized within 0.1 μm of the inner membrane. Using immunofluorescence microscopy, Juarez and co-workers found that VHb is distributed in the cytoplasm and the membranes of organelles in CHO cells [59]. Because periplasmic localization of VHb would enhance the effect of VHb on the electron transport chain, VHb was fused to OmpA and expressed in *E. coli* [58]. Approximately 50% of the OmpA-VHb was located in the periplasmic space. However, no improvement of the growth characteristics or oxygen consumption, compared to the unfused VHb expression, were observed. The authors speculated that cytoplasmic location of VHb may provide an oxygen buffer to facilitate oxygen delivery to the terminal oxidase that are oriented toward the cytoplasm. The authors also mentioned that non-functional apoprotein could have accumulated in the periplasmic space, explaining the lack of effects observed. More recently, the twin-arginine translocase pathway was used to export active VHb into the periplasmic space of *Halomonas bluephagenesis*. This improved cell formation and poly (3-hydroxybutyrate) production under oxygen limitation [60]. Moreover, the authors shown that the intracellular and periplasmic VHb expression increased the amount of proteins related to aerobic respiration in *H. bluephagenesis*, particularly cytochromes and beta subunit of the ATP synthase. Therefore, the presence of VHb affects respiratory efficiency not only by increasing oxygen transport, but also by increasing a higher amount of enzymes of the respiratory chain. This contributes to a better understanding of the enhanced respiratory capacity of cells expressing VHb.

In comparison with the relatively abundant information on the use of VHb for improving bioprocesses under oxygen limitation, the application of VHb for aerobic cultures is scarce. Table 2 shows examples of VHb expression under fully aerobic conditions and the reported effects. Particular physiological responses depend on the host organism and even on the strain used. However, a common factor of the different reports is that higher oxygen uptake and ATP generation can benefit the culture performance. A key aspect to be addressed is the impact of VHb on the overflow metabolism of cells. There are few but relevant reports showing that aerobic VHb expression, in fact, reduces overflow metabolism (for instance in *E. coli* and CHO cells, Table 2). Therefore, VHb expression could be an efficient and simple strategy to overcome overflow metabolism. Potential metabolic reasons for this are discussed below.

### 2.2. Metabolic Consequences of Aerobic Expression of Vitreoscilla Haemoglobin

As explained before, overflow metabolism has been associated with fast growth, since under fast growth energy is generated via fermentation instead of respiration. In addition to bacteria and fungi many other organisms—mammalian cells, plants—use respiration at low glucose uptake rates and aerobic fermentation at high glucose uptake rates [73]. It was shown that NAD^+^/NADH ratio is key to the metabolic differences between the metabolic switches: redox balance is one of the factors leading to overflow metabolism [19,44,74]. As a consequence, cofactor redox engineering strategies have been developed for industrial applications [75].

Using a network-based approach a recent study suggested that there is an upper limit to the Gibbs energy release rate of *E. coli* and *S. cerevisiae* [76]. Due to the different reactions in fermentative pathways and oxidative phosphorylation, thermodynamic driving force in terms of Gibbs energy change is different between those. The authors suggested that this limit in thermodynamics of metabolism might explain the overflow metabolism, since they observed that flux distributions were shifted from respiratory pathways to fermentative pathways with increasing substrate uptake rates.

Through a kinetic model of *E. coli* it was suggested that overflow metabolism should be considered a reversible process and be universal including ethanol consumption by *S. cerevisiae* and lactate by mammalian cells [77]. Mori et al. [78] combined genome-scale modelling with experimental data to characterize yield-cost trade-off in *E. coli* and found that the efficiency of ATP synthesis is the key driver.

Fifteen models of overflow metabolism have been reviewed recently [79]. In addition to the above mentioned constraints of free energy dissipation [76], total proteome [31,79], membrane occupancy [45,46]; the constraints such as electron transfer capacity, oxygen uptake rate, and macromolecular density were observed to be used. Overflow metabolism was also interpreted from the perspective of the regulation of oxidative stress [80] since growth increases as a response to oxidative stress. The trade-off between glucose uptake rate and growth yield was related to the changes in P/O ratio and the flux allocation between glycolysis and pentose phosphate pathway [81]. It has been demonstrated that the presence of VHb in *E. coli* enhanced by 5-fold the content of the cytochrome *bo* and by 1.5 –fold the content of the cytochrome *bd* in cells of *E. coli* [82]. Cytochrome O works as a proton pump, mobilizing 2 protons per electron (H^+^/*e*^−^ = 2). In contrast, an H^+^/*e*^−^ ratio of 1 is obtained by cytochrome *bd* [83]. Therefore, it is reasonable to expect that the presence of VHb alters the P/O ratio.

Here, to investigate the effect of P/O ratio on the overflow metabolism, flux balance analysis (FBA) was carried out using *E. coli* genome scale model [84] in COBRA toolbox [85]. Following the approach in Taymaz-Nikerel et al. [86], the P/O ratio, was varied by modifying the stoichiometry of the reactions catalysed by NADH dehydrogenase (NADH10, NADH16pp, NADH17pp, NADH18pp), FADH dehydrogenase (FDH4pp) and cytochrome oxidases (CYTBD2pp, CYTBDpp, CYTBO3_4pp). Table 3 summarizes the implemented stoichiometric coefficients.

**Figure 2:**
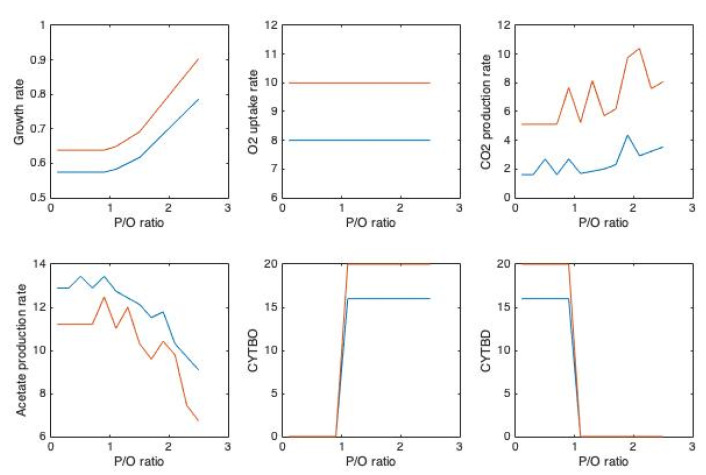
Distribution of Fluxes with Varying P/O ratio. Biomass production is Maximized, Constraints *q_S_* = −11 mmol g^−1^h^−1^ [88], *q_O_*_2_ = −8 mmol g^−1^h^−1^ for wild type (blue) and −8 × 1.25 mmol g^−1^h^−1^ in the presence of VHb (red).

**Figure 3:**
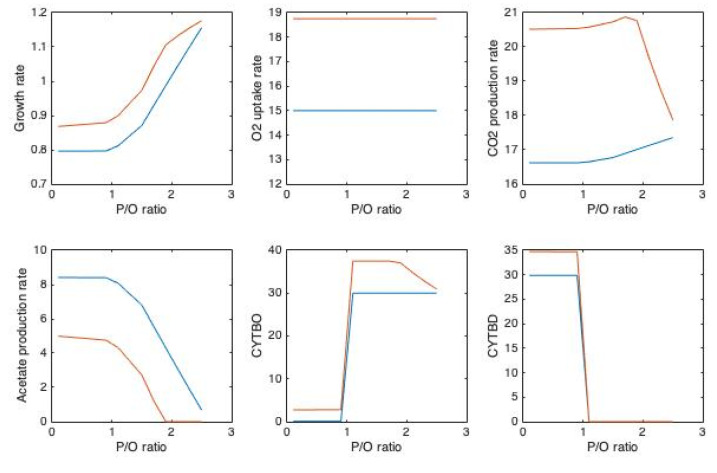
Distribution of fluxes with varying P/O ratio. Biomass production is maximized, constraints *q_S_* = −11 mmol g^−1^h^−1^, *q_O_*_2_ = −15 mmol g^−1^h^−1^ [89] for wild type (blue) and −15 × 1.25 mmol g^−1^h^−1^ in the presence of VHb (red).

Theoretically P/O ratio in *E. coli* varies between 0.67 and 2.67 [87]. For constraints of FBA, maximum glucose uptake rate of *E. coli*, namely *q_S_* = −11 mmol g^−1^h^−1^ [88] and *q_O_*_2_ = −8 mmol g^−1^h^−1^ values were implemented for wild type (Figure 2). For the second case addition to maximum glucose uptake rate of *q_S_* = −11 mmol g^−1^h^−1^, maximum O_2_ uptake rate of *q_O_*_2_ = −15 mmol g^−1^h^−1^ [89] were used (Figure 3) for the wild type. In the presence of VHb cases, addition to the same *q_S_* values, 25% higher O_2_ uptake rates were set due to the experimental measurements [66]. The values for *q_O_*_2_ was selected to cover from relatively low to maximum capacity of the cell. To see the effect of P/O ratio on overflow metabolism, runs for varying P/O were carried out. At low P/O ratio values, acetate production rate was higher, in agreement with the finding that decreased P/O ratios yielded in higher rate of acetate production [81]. In all the cases studied/simulated bd-type cytochrome oxidases replace bo-type for P/O > 1. This might be related with the observation that the fraction of ATP produced by the electron transport chain is higher at high P/O ratios [46]. When q_O2_ is at its maximum value (Figure 3) in the presence of VHb, *q_CO_*_2_ decreases after P/O ratio of around 2.

Figure 2 and Figure 3 clearly show the effect of P/O ratio on the overflow metabolism as acetate production decreases by increasing P/O ratio, consistent with the work of Szenk et al. 2017 [46]. At a fixed *q_O_*_2_, growth rate is maximized at stable *µ* below P/O ratio of 1. For P/O ratio > 1 maximum growth rate increases with increasing P/O ratio. bo-type and bd-type cytochrome oxidases replace each other for P/O ratio below 1 and above 1.

In the presence of VHb, it was observed that *q_O_*_2_ was around 20% higher [66] compared to that in cells without VHb. Acetate production is lower when VHb is present, consistent with previous experimental findings [65] and even totally diminishes at higher *q_O_*_2_ (Figure 3). This implies possibilities for modification of aerobic respiratory chains of organisms to change P/O ratio (different ATP yields) and thus lower the impacts of overflow metabolism, although the complex responses of the organism(s) should not be avoided. On the other hand, it has already been mentioned that the periplasmic proteome of cells can change in response to VHb expression. This may be an adaptation to a presumably crowded section of the cell in order to increase energy production.

These simulations may help to understand how VHb could contribute to reduce production of overflow metabolites. Considerable investigations are still needed to better understand the physiological changes caused by aerobic VHb expression. Nevertheless, the current available information point to VHb technology as a strong ally to improve aerobic bioprocesses.

## Figures and Tables

**Table 1:** Overflow metabolites in several organisms of industrial relevance.

Organism	Main Overflow Metabolites
*Bacillus subtilis*	Acetoin, acetate [9]
CHO cells	Lactate [10]
*Clostridium thermocellum*	Lactate, acetate, ethanol [11]
*Corynebacterium glutamicum*	Dihydroxyacetone, acetate [12]
*Escherichia coli*	Acetate [13]
*Lachance kluyveri*	Ethylacetate [14]
*Saccharomyces cerevisiae*	Ethanol [13]
*Penicillium chrysogenum*	Gluconate [15]
*Pichia pastoris*	Ethanol, acetate [16]

**Table 2:** Reported Effects of *Vitreoscilla* Haemoglobin in Aerobic Cultures.

Organism	Reported Effect
*Aurantiochytrium* sp.	44% higher total fatty acid and 9-fold higher astaxanthin contents [61]
CHO cells	40–100% increase of tissue plasminogen activator production [62] *μ* and biomass yields increase, lactate production per cell decreased by 40% [62] NAD^+^/NADH ratio and ATP cell content decreased, NADP^+^/NADPH ratio increased [59]
*Corynebacterium glutamicum*	Synthesis of biomass increased 10% and L-glutamate production increased 30% [63]
*Escherichia coli*	Increased *q_O_*_2_, *μ* and L-phenylalanine production [64] 60% decrease of acetate accumulation when VHb was expressed from a plasmid. Two-fold increase of plasmid DNA yield from biomass in strain W3110 [65] 37% and 50% reduction in acetate production rate in strains W3110 and BL21, respectively, when VHb was expressed from the chromosome. Different impact on the expression of genes from the TCA cycle and cytochromes, depending on the strain (W3110 or BL21) [66]
*Gluconobacter oxydans*	8% increase of volumetric oxidation activity of N-2-hydroxyethyl glucamine [67]
*Mortierella alpina*	Increased *μ* and 1.6-fold higher arachidonic acid production [68]
*Schwanniomyces occidentalis*	*μ* and alpha-amylase production increased [69]
*Pichia pastoris*	4-fold higher β-galactosidase activity [70] 31.5% higher expression of *Y. lipolytica* LIP2 lipase [71]
*Yarrowia lipolytica*	23% higher *μ*, 2.6-fold higher biomass formation, 92% higher RNase production [72]

**Table 3:** Stoichiometry of dehydrogenases and cytochrome oxidases involved in the *E. coli* metabolic network [84].

Name of the Reaction		Stoichiometry
NADH dehydrogenase	NADH16pp	(2×P/O+1) h[c] + nadh[c] + q8[c] → nad[c] + q8h2[c] +(2×P/O) h[p]
FADH dehydrogenase	FDH4pp	(2×P/O+1) h[c] + q8[c] + for[p] → q8h2[c] + co2[p] + (2×P/O) h[p]
Cytochrome oxidase bd-type	CYTBD2pp	(P/O) h[c] + 0.5 o2[c] + mql8[c] → h2o[c] + mqn8[c] + (P/O) h[p]
Cytochrome oxidase bd-type	CYTBDpp	(P/O) h[c] + 0.5 o2[c] + q8h2[c] → h2o[c] + q8[c] + (P/O) h[p]
Cytochrome oxidase bo-type	CYTBO3_4pp	(2×P/O) h[c] + 0.5 o2[c] + q8h2[c] → h2o[c] + q8[c] +(2×P/O) h[p]

## References

[1] PORTER J.R. (1961). Louis PASTEUR; achievements and disappointments, 1861. Bacteriol. Rev..

[2] Fletcher W.M. (1907). Lactic acid in amphibian muscle. J. Physiol..

[3] Warburg O., Minami S. (1923). Versuche an überlebendem carcinom-gewebe. Klin. Wochenschr..

[4] Crabtree H.G. (1928). The carbohydrate metabolism of certain pathological overgrowths. Biochem. J..

[5] Vazquez A. (2018). Overflow Metabolism: From Yeast to Marathon Runners.

[6] Holms W.H. (1986). The central metabolic pathways of Escherichia coli: Relationship between flux and control at a branch point, efficiency of conversion to biomass, and excretion of acetate. Curr. Top Cell. Regul..

[7] De Mey M., De Maeseneire S., Soetaert W., Vandamme E. (2007). Minimizing acetate formation in E. coli fermentations. J. Ind. Microbiol. Biotechnol..

[8] Eiteman M.A., Altman E. (2006). Overcoming acetate in Escherichia coli recombinant protein fermentations. Trends Biotechnol..

[9] Ma W., Liu Y., Lv X., Li J., Du G., Liu L. (2019). Combinatorial pathway enzyme engineering and host engineering overcomes pyruvate overflow and enhances overproduction of N-acetylglucosamine in Bacillus subtilis. Microb. Cell Fact..

[10] Luo J., Vijayasankaran N., Autsen J., Santuray R., Hudson T., Amanullah A., Li F. (2012). Comparative metabolite analysis to understand lactate metabolism shift in Chinese hamster ovary cell culture process. Biotechnol. Bioeng..

[11] Thompson R.A., Trinh C.T. (2017). Overflow metabolism and growth cessation in Clostridium thermocellum DSM1313 during high cellulose loading fermentations. Biotechnol. Bioeng..

[12] Wittmann C., Kiefer P., Zelder O. (2004). Metabolic fluxes in Corynebacterium glutamicum during lysine production with sucrose as carbon source. Appl. Environ. Microbiol..

[13] Paczia N., Nilgen A., Lehmann T., Gätgens J., Wiechert W., Noack S. (2012). Extensive exometabolome analysis reveals extended overflow metabolism in various microorganisms. Microb. Cell Fact..

[14] Nanda P., Patra P., Das M., Ghosh A. (2020). Reconstruction and analysis of genome-scale metabolic model of weak Crabtree positive yeast Lachancea kluyveri. Sci. Rep..

[15] Schmitz K., Peter V., Meinert S., Kornfeld G., Hardiman T., Wiechert W., Noack S. (2013). Simultaneous utilization of glucose and gluconate in Penicillium chrysogenum during overflow metabolism. Biotechnol. Bioeng..

[16] Nocon J., Steiger M.G., Pfeffer M., Sohn S.B., Kim T.Y., Maurer M., Rußmayer H., Pflügl S., Ask M., Haberhauer-Troyer C. (2014). Model based engineering of Pichia pastoris central metabolism enhances recombinant protein production. Metab. Eng..

[17] Majewski R.A., Domach M.M. (1990). Simple constrained-optimization view of acetate overflow in E. coli. Biotechnol. Bioeng..

[18] Kayser A., Weber J., Hecht V., Rinas U. (2005). Metabolic flux analysis of Escherichia coli in glucose-limited continuous culture. I. Growth-rate-dependent metabolic efficiency at steady state. Microbiology.

[19] Vemuri G.N., Altman E., Sangurdekar D.P., Khodursky A.B., Eiteman M.A. (2006). Overflow metabolism in Escherichia coli during steady-state growth: Transcriptional regulation and effect of the redox ratio. Appl. Environ. Microbiol..

[20] Yeo H.C., Hong J., Lakshmanan M., Lee D.Y. (2020). Enzyme capacity-based genome scale modelling of CHO cells. Metab. Eng..

[21] Shojaosadati S.A., Kolaei S.M.V., Babaeipour V., Farnoud A.M. (2008). Recent advances in high cell density cultivation for production of recombinant protein. Iran. J. Biotechnol..

[22] De Anda R., Lara A.R., Hernández V., Hernández-Montalvo V., Gosset G., Bolívar F., Ramírez O.T. (2006). Replacement of the glucose phosphotransferase transport system by galactose permease reduces acetate accumulation and improves process performance of Escherichia coli for recombinant protein production without impairment of growth rate. Metab. Eng..

[23] Fuentes L.G., Lara A.R., Martínez L.M., Ramírez O.T., Martínez A., Bolívar F., Gosset G. (2013). Modification of glucose import capacity in Escherichia coli: Physiologic consequences and utility for improving DNA vaccine production. Microb. Cell Fact..

[24] Negrete A., Majdalani N., Phue J.N., Shiloach J. (2013). Reducing acetate excretion from E. coli K-12 by over-expressing the small RNA SgrS. New Biotechnol..

[25] Bäcklund E., Markland K., Larsson G. (2008). Cell engineering of Escherichia coli allows high cell density accumulation without fed-batch process control. Bioprocess Biosyst. Eng..

[26] Wlaschin K.F., Hu W.S. (2007). Engineering cell metabolism for high-density cell culture via manipulation of sugar transport. J. Biotechnol..

[27] Veit A., Polen T., Wendisch V.F. (2007). Global gene expression analysis of glucose overflow metabolism in Escherichia coli and reduction of aerobic acetate formation. Appl. Microbiol. Biotechnol..

[28] Vemuri G.N., Eiteman M.A., McEwen J.E., Olsson L., Nielsen J. (2007). Increasing NADH oxidation reduces overflow metabolism in Saccharomyces cerevisiae. Proc. Natl. Acad. Sci. USA.

[29] Bulté D.B., Palomares L.A., Parra C.G., Martínez J.A., Contreras M.A., Noriega L.G., Ramírez O.T. (2020). Overexpression of the mitochondrial pyruvate carrier reduces lactate production and increases recombinant protein productivity in CHO cells. Biotechnol. Bioeng..

[30] Peebo K., Valgepea K., Maser A., Nahku R., Adamberg K., Vilu R. (2015). Proteome reallocation in Escherichia coli with increasing specific growth rate. Mol. Biosyst..

[31] Basan M., Hui S., Okano H., Zhang Z., Shen Y., Williamson J.R., Hwa T. (2015). Overflow metabolism in Escherichia coli results from efficient proteome allocation. Nature.

[32] Zeng H., Yang A. (2019). Modelling overflow metabolism in Escherichia coli with flux balance analysis incorporating differential proteomic efficiencies of energy pathways. BMC Syst. Biol..

[33] Chen Y., Nielsen J. (2019). Energy metabolism controls phenotypes by protein efficiency and allocation. Proc. Natl. Acad. Sci. USA.

[34] Zeng H., Yang A. (2019). Quantification of proteomic and metabolic burdens predicts growth retardation and overflow metabolism in recombinant Escherichia coli. Biotechnol. Bioeng..

[35] Chen Y., van Pelt-KleinJan E., van Olst B., Douwenga S., Boeren S., Bachmann H., Molenaar D., Nielsen J., Teusink B. (2021). Proteome constraints reveal targets for improving microbial fitness in nutrient-rich environments. Mol. Syst. Biol..

[36] Liu J.K., Lloyd C., Al-Bassam M.M., Ebrahim A., Kim J.N., Olson C., Aksenov A., Dorrestein P., Zengler K. (2019). Predicting proteome allocation, overflow metabolism, and metal requirements in a model acetogen. PLoS Comput. Biol..

[37] Lastiri-Pancardo G., Mercado-Hernández J.S., Kim J., Jiménez J.I., Utrilla J. (2020). A quantitative method for proteome reallocation using minimal regulatory interventions. Nat. Chem. Biol..

[38] de la Cruz M., Ramírez E.A., Sigala J.C., Utrilla J., Lara A.R. (2020). Plasmid DNA Production in Proteome-Reduced. Microorganisms.

[39] Brown G.C. (1991). Total cell protein concentration as an evolutionary constraint on the metabolic control distribution in cells. J. Theor. Biol..

[40] Vazquez A., Oltvai Z.N. (2016). Macromolecular crowding explains overflow metabolism in cells. Sci. Rep..

[41] Beg Q.K., Vazquez A., Ernst J., de Menezes M.A., Bar-Joseph Z., Barabási A.L., Oltvai Z.N. (2007). Intracellular crowding defines the mode and sequence of substrate uptake by Escherichia coli and constrains its metabolic activity. Proc. Natl. Acad. Sci. USA.

[42] Vazquez A., Beg Q.K., Demenezes M.A., Ernst J., Bar-Joseph Z., Barabási A.L., Boros L.G., Oltvai Z.N. (2008). Impact of the solvent capacity constraint on E. coli metabolism. BMC Syst. Biol..

[43] Vazquez A., Liu J., Zhou Y., Oltvai Z.N. (2010). Catabolic efficiency of aerobic glycolysis: The Warburg effect revisited. BMC Syst. Biol..

[44] van Hoek M.J., Merks R.M. (2012). Redox balance is key to explaining full vs. partial switching to low-yield metabolism. BMC Syst. Biol..

[45] Zhuang K., Vemuri G.N., Mahadevan R. (2011). Economics of membrane occupancy and respiro-fermentation. Mol. Syst. Biol..

[46] Szenk M., Dill K.A., de Graff A.M.R. (2017). Why Do Fast-Growing Bacteria Enter Overflow Metabolism? Testing the Membrane Real Estate Hypothesis. Cell Syst..

[47] Stark B.C., Pagilla K.R., Dikshit K.L. (2015). Recent applications of Vitreoscilla hemoglobin technology in bioproduct synthesis and bioremediation. Appl. Microbiol. Biotechnol..

[48] Efiok B.J., Webster D.A. (1990). A cytochrome that can pump sodium ion. Biochem. Biophys. Res. Commun..

[49] Stark B.C., Dikshit K.L., Pagilla K.R. (2012). The Biochemistry of Vitreoscilla hemoglobin. Comput. Struct. Biotechnol. J..

[50] Stark B.C., Dikshit K.L., Pagilla K.R. (2011). Recent advances in understanding the structure, function, and biotechnological usefulness of the hemoglobin from the bacterium Vitreoscilla. Biotechnol. Lett..

[51] Yu F., Zhao X., Wang Z., Liu L., Yi L., Zhou J., Li J., Chen J., Du G. (2021). Recent Advances in the Physicochemical Properties and Biotechnological Application of. Microorganisms.

[52] Webster D.A., Dikshit K.L., Pagilla K.R., Stark B.C. (2021). The Discovery of Vitreoscilla Hemoglobin and Early Studies on Its Biochemical Functions, the Control of Its Expression, and Its Use in Practical Applications. Microorganisms.

[53] Dikshit R.P., Dikshit K.L., Liu Y.X., Webster D.A. (1992). The bacterial hemoglobin from Vitreoscilla can support the aerobic growth of Escherichia coli lacking terminal oxidases. Arch. Biochem. Biophys..

[54] Dikshit K.L., Webster D.A. (1988). Cloning, characterization and expression of the bacterial globin gene from Vitreoscilla in Escherichia coli. Gene.

[55] Kallio P.T., Kim D.J., Tsai P.S., Bailey J.E. (1994). Intracellular expression of Vitreoscilla hemoglobin alters Escherichia coli energy metabolism under oxygen-limited conditions. Eur. J. Biochem..

[56] Jaén K.E., Velazquez D., Delvigne F., Sigala J.C., Lara A.R. (2019). Engineering, E. coli for improved microaerobic pDNA production. Bioprocess Biosyst. Eng..

[57] Khosla C., Bailey J.E. (1989). Evidence for partial export of Vitreoscilla hemoglobin into the periplasmic space in Escherichia coli. Implications for protein function. J. Mol. Biol..

[58] Ramandeep, Hwang K.W., Raje M., Kim K.J., Stark B.C., Dikshit K.L., Webster D.A. (2001). Vitreoscilla hemoglobin. Intracellular localization and binding to membranes. J. Biol. Chem..

[59] Juárez M., González-De la Rosa C.H., Sigala J.C., Lara A.R. (2021). Effect of *V**itreoscilla* hemoglobin on recombinant protein expression and energy metabolism of CHO cells. Rev. Mex. Ing. Quím..

[60] Ouyang P., Wang H., Hajnal I., Wu Q., Guo Y., Chen G.Q. (2018). Increasing oxygen availability for improving poly(3-hydroxybutyrate) production by Halomonas. Metab. Eng..

[61] Suen Y.L., Tang H., Huang J., Chen F. (2014). Enhanced production of fatty acids and astaxanthin in Aurantiochytrium sp. by the expression of Vitreoscilla hemoglobin. J. Agric. Food Chem..

[62] Juárez M., González-De la Rosa C.H., Memún E., Sigala J.C., Lara A.R. (2017). Aerobic expression of Vitreoscilla hemoglobin improves the growth performance of CHO-K1 cells. Biotechnol. J..

[63] Liu Q., Zhang J., Wei X.X., Ouyang S.P., Wu Q., Chen G.Q. (2008). Microbial production of L -glutamate and L -glutamine by recombinant Corynebacterium glutamicum harboring Vitreoscilla hemoglobin gene vgb. Appl. Microbiol. Biotechnol..

[64] Wu W.B., Guo X.L., Zhang M.L., Huang Q.G., Qi F., Huang J.Z. (2018). Enhancement of l-phenylalanine production in Escherichia coli by heterologous expression of Vitreoscilla hemoglobin. Biotechnol. Appl. Biochem..

[65] Pablos T.E., Sigala J.C., Le Borgne S., Lara A.R. (2014). Aerobic expression of Vitreoscilla hemoglobin efficiently reduces overflow metabolism in Escherichia coli. Biotechnol. J..

[66] Lara A.R., Galindo J., Jaén K.E., Juárez M., Sigala J.C. (2020). Physiological Response of. J. Microbiol. Biotechnol..

[67] Liu D., Ke X., Hu Z.C., Zheng Y.G. (2021). Improvement of pyrroloquinoline quinone-dependent d-sorbitol dehydrogenase activity from Gluconobacter oxydans via expression of Vitreoscilla hemoglobin and regulation of dissolved oxygen tension for the biosynthesis of 6-(N-hydroxyethyl)-amino-6-deoxy-α-l-sorbofuranose. J. Biosci. Bioeng..

[68] Zhang H., Feng Y., Cui Q., Song X. (2017). Expression of Vitreoscilla hemoglobin enhances production of arachidonic acid and lipids in Mortierella alpina. BMC Biotechnol..

[69] Suthar D.H., Chattoo B.B. (2006). Expression of Vitreoscilla hemoglobin enhances growth and levels of alpha-amylase in Schwanniomyces occidentalis. Appl. Microbiol. Biotechnol..

[70] Wu J.M., Hsu T.A., Lee C.K. (2003). Expression of the gene coding for bacterial hemoglobin improves beta-galactosidase production in a recombinant Pichia pastoris. Biotechnol. Lett..

[71] Wang X., Sun Y., Shen X., Ke F., Zhao H., Liu Y., Xu L., Yan Y. (2012). Intracellular expression of Vitreoscilla hemoglobin improves production of Yarrowia lipolytica lipase LIP2 in a recombinant Pichia pastoris. Enzyme. Microb. Technol..

[72] Bhave S.L., Chattoo B.B. (2003). Expression of vitreoscilla hemoglobin improves growth and levels of extracellular enzyme in Yarrowia lipolytica. Biotechnol. Bioeng..

[73] Huberts D.H., Niebel B., Heinemann M. (2012). A flux-sensing mechanism could regulate the switch between respiration and fermentation. FEMS Yeast Res..

[74] Pinu F.R., Granucci N., Daniell J., Han T.L., Carneiro S., Rocha I., Nielsen J., Villas-Boas S.G. (2018). Metabolite secretion in microorganisms: The theory of metabolic overflow put to the test. Metabolomics.

[75] Liu J., Li H., Zhao G., Caiyin Q., Qiao J. (2018). Redox cofactor engineering in industrial microorganisms: Strategies, recent applications and future directions. J. Ind. Microbiol. Biotechnol..

[76] Niebel B., Leupold S., Heinemann M. (2019). An upper limit on Gibbs energy dissipation governs cellular metabolism. Nat. Metab..

[77] Millard P., Enjalbert B., Uttenweiler-Joseph S., Portais J.C., Létisse F. (2021). Control and regulation of acetate overflow in Escherichia coli. Elife.

[78] Mori M., Marinari E., De Martino A. (2019). A yield-cost tradeoff governs Escherichia coli’s decision between fermentation and respiration in carbon-limited growth. NPJ Syst. Biol. Appl..

[79] de Groot D.H., Lischke J., Muolo R., Planqué R., Bruggeman F.J., Teusink B. (2020). The common message of constraint-based optimization approaches: Overflow metabolism is caused by two growth-limiting constraints. Cell Mol. Life Sci..

[80] Shimizu K., Matsuoka Y. (2019). Redox rebalance against genetic perturbations and modulation of central carbon metabolism by the oxidative stress regulation. Biotechnol. Adv..

[81] Cheng C., O’Brien E.J., McCloskey D., Utrilla J., Olson C., LaCroix R.A., Sandberg T.E., Feist A.M., Palsson B.O., King Z.A. (2019). Laboratory evolution reveals a two-dimensional rate-yield tradeoff in microbial metabolism. PLoS Comput. Biol..

[82] Tsai P.S., Nägeli M., Bailey J.E. (1996). Intracellular expression of Vitreoscilla hemoglobin modifies microaerobic Escherichia coli metabolism through elevated concentration and specific activity of cytochrome o. Biotechnol. Bioeng..

[83] Puustinen A., Finel M., Haltia T., Gennis R.B., Wikström M. (1991). Properties of the two terminal oxidases of Escherichia coli. Biochemistry.

[84] Orth J.D., Conrad T.M., Na J., Lerman J.A., Nam H., Feist A.M., Palsson B. (2011). A comprehensive genome-scale reconstruction of Escherichia coli metabolism--2011. Mol. Syst. Biol..

[85] Schellenberger J., Que R., Fleming R., Thiele I., Orth J., Feist A., Zielinski D., Bordbar A., Lewis N., Rahmanian S. (2011). Quantitative prediction of cellular metabolism with constraint-based models: The COBRA Toolbox v2.0. Nat. Protoc..

[86] Taymaz-Nikerel H., Borujeni A.E., Verheijen P.J., Heijnen J.J., van Gulik W.M. (2010). Genome-derived minimal metabolic models for Escherichia coli MG1655 with estimated in vivo respiratory ATP stoichiometry. Biotechnol. Bioeng..

[87] Kalnenieks U., Balodite E., Rutkis R. (2019). Metabolic Engineering of Bacterial Respiration: High vs. Low P/O and the Case of. Front. Bioeng. Biotechnol..

[88] Fischer E., Zamboni N., Sauer U. (2004). High-throughput metabolic flux analysis based on gas chromatography-mass spectrometry derived 13C constraints. Anal. Biochem..

[89] Carlson R., Srienc F. (2004). Fundamental Escherichia coli biochemical pathways for biomass and energy production: Creation of overall flux states. Biotechnol. Bioeng..

